# Ultrasensitive and Multiple Biomarker Discrimination for Alzheimer's Disease via Plasmonic & Microfluidic Sensing Technologies

**DOI:** 10.1002/advs.202308783

**Published:** 2024-03-20

**Authors:** Lijiao Zu, Xicheng Wang, Peng Liu, Jiwei Xie, Xuejun Zhang, Weiru Liu, Zhencheng Li, Shiqing Zhang, Kaiwei Li, Ambra Giannetti, Wei Bi, Francesco Chiavaioli, Lei Shi, Tuan Guo

**Affiliations:** ^1^ Institute of Photonics Technology Jinan University Guangzhou 510632 China; ^2^ State Key Laboratory of Bioactive Molecules and Druggability Assessment JNU‐HKUST Joint Laboratory for Neuroscience and Innovative Drug Research, College of Pharmacy, Jinan University Guangzhou 510632 China; ^3^ Center for Advanced Biomedical Imaging and Photonics, Division of Gastroenterology, Department of Medicine Beth Israel Deaconess Medical Center, Harvard University Boston 02215 USA; ^4^ National Research Council of Italy (CNR), Institute of Applied Physics “Nello Carrara” (IFAC) Sesto Fiorentino 50019 Italy; ^5^ Department of Neurology The First Affiliated Hospital of Jinan University Guangzhou 510632 China

**Keywords:** Alzheimer's disease, microfluidics, monomer and oligomer of Aβ_42_, surface plasmon resonance, tilted fiber Bragg grating

## Abstract

As the population ages, the worldwide prevalence of Alzheimer's disease (AD) as the most common dementia in the elderly is increasing dramatically. However, a long‐term challenge is to achieve rapid and accurate early diagnosis of AD by detecting hallmarks such as amyloid beta (Aβ_42_). Here, a multi‐channel microfluidic‐based plasmonic fiber‐optic biosensing platform is established for simultaneous detection and differentiation of multiple AD biomarkers. The platform is based on a gold‐coated, highly‐tilted fiber Bragg grating (TFBG) and a custom‐developed microfluidics. TFBG excites a high‐density, narrow‐cladding‐mode spectral comb that overlaps with the broad absorption of surface plasmons for high‐precision interrogation, enabling ultrasensitive monitoring of analytes. In situ detection and in‐parallel discrimination of different forms of Aβ_42_ in cerebrospinal fluid (CSF) are successfully demonstrated with a detection of limit in the range of ≈30–170 pg mL^−1^, which is one order of magnitude below the clinical cut‐off level in AD onset, providing high detection sensitivity for early diagnosis of AD. The integration of the TFBG sensor with multi‐channel microfluidics enables simultaneous detection of multiple biomarkers using sub‐µL sample volumes, as well as combining initial binding rate and real‐time response time to differentiate between multiple biomarkers in terms of binding kinetics. With the advantages of multi‐parameter, low consumption, and highly sensitive detection, the sensor represents an urgently needed potentials for large‐scale diagnosis of diseases at early stage.

## Graphical abstract

The combination of highly‐sensitive plasmonic‐enhanced fiber‐optic devices with multi‐channel microfluidics enables the simultaneous analysis of multiple biomarkers using sub‐microliter sample volumes. The biophotonic platform developed endows the detection and discrimination of different forms of Aβ_42_ (monomer and oligomer) in cerebrospinal fluid, as Alzheimer's disease hallmark at early stage, thus paving the way for large‐scale diagnosis of the onset of diseases.

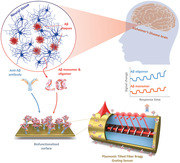

## 1 Introduction

Alzheimer's disease (AD), characterized by progressive amnestic syndrome, is the most common neurodegenerative disease and becomes a major risk to human health.^[^ ^1^ ^]^ Over 55 million people are living with dementia worldwide in 2021, and the number is expected to reach to 78 million by 2030.^[^ ^2^ ^]^ AD is the most prevalent form of dementia and contributes to about 60–70% of the cases,^[^ ^3^ ^]^ and the burden of AD is expected to continuously increase with the population aging. However, there is currently no cure for AD due to its intricate pathogenesis.^[^ ^4^ ^]^ Thus, early diagnosis of AD and early intervention before the appearance of overt clinical symptoms is critically required to prevent AD progression.^[^ ^5^ ^]^

Currently, according to the guidelines of the National Institute on Aging and Alzheimer's Association, the framework for AD diagnosis relies on the classification of individuals based on the so‐called ATN system (β‐Amyloid (Aβ), hyperphosphorylated Tau and Neurodegeneration), which is a combination of biomarkers in cerebrospinal fluid (CSF) with neuroimaging techniques (positron emission tomography and magnetic resonance imaging), in addition to other cognitive and psychological assessments.^[^ ^6^, ^7^, ^8^, ^9^ ^]^ Regarding the pathogenesis of AD, the Aβ cascade hypothesis has been the dominant hypothesis for more than three decades, which suggests that the deposition of Aβ in the brain is the centerpiece of AD pathology and leads to cognitive deficits in patients.^[^ ^10^, ^11^ ^]^ Although this hypothesis is still controversial.^[^ ^12^ ^]^ the recent successes with Aβ‐targeting monoclonal antibody for AD treatment indicate that Aβ is crucial for AD pathogenesis.^[^ ^13^, ^14^ ^]^ The Aβ levels in CSF are currently considered as the effective biomarker for early diagnosis of mild cognitive impairment and AD.^[^ ^15^, ^16^ ^]^ The Aβ is derived from the cleavage of the amyloid precursor protein by the enzymes β‐secretase and γ‐secretase.^[^ ^17^, ^18^ ^]^ and consists mainly of Aβ_40_ (40 amino acids) and Aβ_42_ (42 amino acids).^[^ ^19^ ^]^ Studies have demonstrated that with the progression of AD, the change of Aβ_42_ levels in CSF showed significantly higher discriminative power than Aβ_40_ levels in identifying 18F‐flutemetamol‐positive patients.^[^ ^20^, ^21^ ^]^ and the detection of Aβ_42_‐related indexes, such as Aβ_42_ alone, Aβ_42_/Aβ_40_, and p‐Tau/Aβ_42_ in CSF, is a reliable indicator for the early diagnosis of AD.^[^ ^7^, ^22^, ^23^, ^24^ ^]^ It was found that the sensitivity and specificity of AD diagnosis based on Aβ_42_ detection were 85% and 95%, respectively, highlighting the importance of Aβ_42_ in the diagnosis of the disease.^[^ ^25^ ^]^ In addition, the Aβ_42_ monomer could spontaneously aggregates into Aβ_42_ oligomers in physiological conditions.^[^ ^26^, ^27^ ^]^ The soluble Aβ_42_ oligomers are more toxic than Aβ_42_ monomers,^[^ ^28^ ^]^ which can exert their neurotoxicity independently of mature fibrils.^[^ ^26^, ^29^ ^]^ and inhibition of Aβ_42_ aggregation is considered as a potential target for anti‐AD drugs development.^[^ ^30^ ^]^ Several studies have confirmed that an increase in Aβ_42_ oligomers or a decrease in Aβ_42_ monomers in CSF are highly associated with the increased risk of AD.^[^ ^31^, ^32^, ^33^ ^]^ The above results suggest that the detection of Aβ_42_, either monomer or oligomer forms, in CSF is an effective strategy of AD diagnosis. However, the traditional methods of fluid biomarker testing are time‐consuming with low sensitivity and accuracy, and may lead to false‐positive diagnoses.^[^ ^34^ ^]^

Recently, many optical biosensors have been developed to quantify AD biomarkers, especially Aβ, for early diagnosis of AD, including those based on fluorescence,^[^ ^35^, ^36^, ^37^ ^]^ colorimetric,^[^ ^38^ ^]^ surface enhanced Raman scattering (SERS),^[^ ^39^ ^]^ lossy‐mode resonance (LMR),^[^ ^40^ ^]^ and surface plasmon resonance (SPR).^[^ ^41^, ^42^, ^43^, ^44^ ^]^ Among the above‐mentioned numerous optical sensors, label‐free optical biosensors, especially fiber‐optic SPR biosensors, have become a research hotspot due to their excellent performances, such as real‐time and direct detection of biological targets with high specificity and sensitivity, small size and low costs. Compared with bulky prism configurations (i.e., Kretschmann–Raether prism),^[^ ^45^ ^]^ fiber‐optic SPR biosensors fabricated from cladding‐modified fibers (unclad, side‐polished, tapered, and U‐shaped) coated with noble metal nanolayers (gold or silver) have attracted great interest from researchers because of their miniaturization, remote operation and ease of use. For example, Truong Thi Vu Nu et al. coated the core surface of a multimode fiber with gold (Au) and then immobilized antibodies on the Au surface to achieve specific detection of Tau, another AD biomarker.^[^ ^46^ ^]^

With the development of fiber‐optic SPR biosensors, tilted fiber Bragg grating (TFBG) based SPR biosensors stand out due to their good sensing performance and special coupling mechanisms. First of all, it can generate dense comb like resonance peaks, including tens or even hundreds of extremely narrow linewidth cladding modes, Ghost mode and Bragg mode, due to the introduction of the tilt angle, providing a very rich mode field distribution and different degrees of response characteristics to the external environmental changes. These features enable to achieve high sensitivity and resolution in the detection; for example, a resolution in refractive index (RI) changes can be as high as 10^−6^ to 10^−8^ refractive index units (RIU).^[^ ^47^ ^]^ Moreover, it also retains the backward transmission to the core mode that meets the Bragg reflection condition. By monitoring the fiber core mode, the parametric cross‐sensitivity can be effectively eliminated during the detection process, thus realizing simultaneous independent partitioning of multiple parametric measurements and improving the sensing accuracy.^[^ ^48^ ^]^ Besides, the mechanical properties of TFBG‐based SPR biosensors are stable and the production technology is mature enough for large‐scale batch production.^[^ ^49^, ^50^ ^]^

Therefore, considering the excellent performance of SPR and the need for early diagnosis of AD, we developed a TFBG‐SPR biosensor to achieve ultra‐high sensitivity in the detection of Aβ_42_ level in CSF. The TFBG‐SPR biosensors were placed in a novel‐designed custom‐made multi‐channel microfluidic system to achieve for the very first time trace detection and discrimination of various biomarkers in parallel. To this aim, an interrogator system that has multiple optical channels for acquisition and data processing was integrated into the experimental setup to enable real‐time detection and portable operation of multiple samples. The covalent surface modification of the TFBG‐SPR biosensors using self‐assembly technology quantifying the specific interaction between Aβ_42_ antibody and Aβ_42_ achieving detection limits in the range of tens of pg mL^−1^, which is a level below the clinical cut‐off value. In addition, the proposed biosensing platform was used to investigate the different forms of Aβ_42_ (monomer and oligomer) by response time, initial binding rate and sensitivity, which can mutually be corroborated with the aggregation process of altered proteins in AD. Finally, the detection specificity was also confirmed using animal CSF samples and injecting non‐specific types of analytes, thus envisaging a reliable and effective biophotonic platform that empowers and supports clinical treatments of diseases at early stages toward personalized medicine.

## 2 Result

### 2.1 Real‐Time Monitoring of Pathological Changes in AD

**Figure 1:**
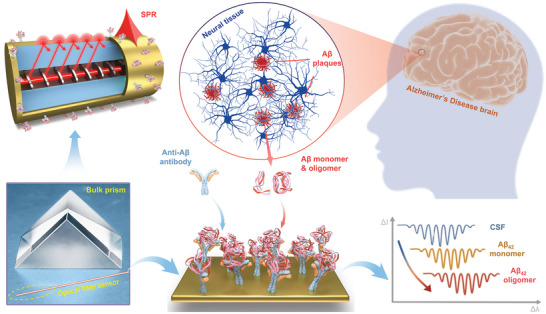
Concept of plasmonic optical fiber sensing for Alzheimer's disease. Schematic illustration of a gold‐coated fiber‐optic sensor immersed in cerebrospinal fluid for real‐time monitoring of pathological processes and biomarker concentrations in Alzheimer's disease.

AD is a slowly developing disease in which neuropathological features begin to emerge in the brain ≈20 years before symptoms appear.^[^ ^51^ ^]^ One of its earliest pathological features is brain deposition of Aβ.^[^ ^52^ ^]^ Under pathological conditions, Aβ forms aggregates from monomers, ranging from soluble oligomers to long amyloid fibrils. Despite intense interest in developing measures of AD‐related parameters at early stage, this progress has been hampered by limited sensitivity and poor correlation with brain pathology. We attempted to develop a plasmonic fiber optic sensing platform for highly sensitive, multiparameter analysis of Aβ in CSF, as shown in **Figure** **1**.

TFBG combined with SPR provides a unique tool by hybridizing the high‐density narrowband spectral comb provided by the core‐to‐cladding resonance mechanism with the broad absorption of surface plasmon polaritons. By measuring the amplitude changes of the grating resonance located on the edge of SPR signature, high‐precision measurement of small shifts of SPR can be achieved. In addition, TFBG‐assisted fiber‐optic SPR works in the near‐infrared band and can provide longer penetration depth and propagation length, thus making it super sensitive to changes near the surface where functional layers and binding events occur. These characteristics promote the development of high‐performance biosensors for disease‐related biomarker detection at very low level, and hence empower an early diagnosis of diseases.

### 2.2 Characteristics of Interface Reactions over Optical Fiber Sensor

**Figure 2:**
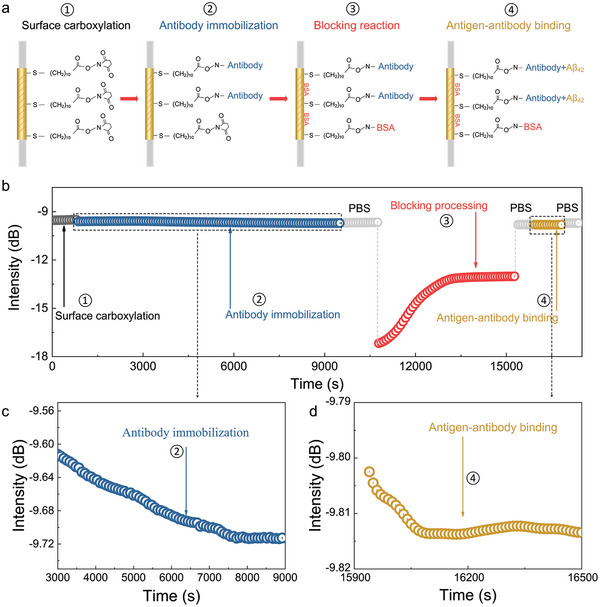
Surface modification processes and their optical responses recorded by optical fiber sensor. a) Schematic illustration of immobilizing the antibody and BSA blocking agent on the fiber surface and then detecting the Aβ_42_; b) the real‐time response curve of the optical biosensor at different steps of the functionalization process, that is, intensity variation as a function of time during the PBS rising, antibody surface activation, and BSA surface passivation, as well as Aβ_42_ detection; c) local enlarged view of the antibody incubation process; d) enlarged view of spectral intensity changes before and after Aβ_42_ detection at a concentration of 0.1 ng mL^−1^.

In order to guarantee both the accuracy in the measurement and the repeatability in the results achieved during the experiments, each sensor is calibrated before detection, and its sensitivity is about 3500 dB/RIU (Figure S1, Supporting Information). Afterward the functionalization of the sensitive region of the fiber surface can be performed, and the step‐by‐step process for the preparation of the sensing bio‐layer is shown in **Figure** **2**a. The fiber coated with a gold layer was functionalized using a self‐assembled monolayer (SAM) of 11‐mercaptoundecanoic acid (MUA). This molecule is commonly used for its capacity to anchor to gold forming AU–S bonds through its thiol‐terminated functional group,^[^ ^47^, ^53^ ^]^ and hence it provides exposed carboxyl groups for effective binding of anti‐Aβ_42_ antibodies (the biological recognition elements; BREs) to the amino group via 1‐(3‐dimethylaminopropyl)‐3‐ethylcarbodiimide hydro (EDC)/*N*‐hydroxy succinimide (NHS) chemistry. Simultaneously, the bovine serum albumin (BSA) solution is incubated to block non‐specific binding sites and passivate the surface,^[^ ^40^, ^54^ ^]^ and hence to enhance the specific interaction of the antibody with Aβ_42_, thereby improving the signal‐to‐noise ratio.^[^ ^47^ ^]^

After the complete immunoassay is performed, a thin biofilm is formed on the surface of the optical fiber. As more biomolecules are bound, the amplitude of the plasmonic‐enhanced cladding modes changes accordingly and all modes exhibit different relative intensity sensitivities. The most sensitive mode was chosen for the relationship between biofilm formation and spectral changes. The real‐time monitoring of biofilm formation process on the surface of TFBG‐SPR shown in Figure 2b. The relative intensity of the selected cladding mode changed significantly from the formation of a thiolated organic molecule film on the surface of TFBG to the antibody immobilization onto the surface, passing by the surface passivation with BSA blocking agent, and finally reaching the binding of Aβ_42_ to the antibody. This demonstrates that the biochemical reaction process on the TFBG‐SPR surface can be monitored in real‐time by the relative intensity changes of the surface plasmonic mode, which provides a new tool and platform for further exploring the small changes in the biochemical reaction process. It is worth underlining that any change in the RI stems from two independent contributions: i) bulk or volume RI change that is related to the RI of the solution and occurs immediately after the injection of the solution, and ii) surface RI change that is related to the amount of biomolecules bound to the surface and can be assessed when the optical signal in running buffer (PBS in this case) is compared before and after the injection of the solution.^[^ ^48^, ^55^ ^]^ Therefore, the greater changes that occurred in step ③ of Figure 2b can be explained by the fact that the RI of BSA is about 1.3345, while the RI of the other biological analytes is about 1.3334 or even lower, and hence more than one order of magnitude smaller than that of BSA. As a result, the bulk RI change measured by the SPR mode was larger than any other; conversely, the surface RI change was very small and almost negligible.

Furthermore, from the real‐time monitoring process, it can be seen that the antibody has been attached onto the TFBG‐SPR surface, which exhibits the following features: i) its spectrum no longer changes with time; ii) the intensity of the spectrum changes before and after the introduction of the antibody, thus testifying the successful attachment of the antibody onto the TFBG‐SPR surface (Figure 2c). What is interesting is that we found that the optical characteristics of the spectrum change when the antibody grafted in the biofilm of the sensor binds to the Aβ_42_ protein to be measured (Figure 2d). The reason for this change can stem from the fact that the Aβ_42_ passes through the microfluidic system, the interaction between the antigen and the antibody occurs, resulting in a change in the dielectric properties (surface charge, steric hindrance, etc.) of the medium on the sensor surface,^[^ ^56^ ^]^ which results in the change in both the effective RI and thickness of the biofilm surrounding the fiber.^[^ ^57^ ^]^ In general, the change in biofilm thickness can be reflected as a change in the surface RI^[^ ^55^ ^]^ caused by the binding of the Aβ_42_ protein to the antibody. Therefore, we quantified those changes caused by different concentrations of Aβ_42_ protein by selecting the corresponding specific intensity patterns.

Above all, the SPR signal generated by the light passing through the biofilm layer of the TFBG‐SPR will be modulated by the thickness of the biofilm layer. Therefore, the concentration of the targeted molecule can be assessed according to the changes (both wavelength and intensity) in the spectral features before and after the binding of the molecule to be measured when the signal baseline is recovered.

### 2.3 In Situ Quantification of Aβ_42_ Monomer and Oligomer in CSF

First, we detected the spectral response of Aβ_42_ protein at different concentrations spiked in PBS. The intensity changes given by Aβ_42_ monomer (red bar chart) and oligomers (blue bar chart), with concentrations increasing from 0.1 to 1000 ng mL^−1^ with a dynamic range of over five orders of magnitude is shown in Figure S2, Supporting Information, which well confirms the feasibility of using TFBG‐SPR biosensor to detect Aβ_42_ monomers and oligomers.

Next, we investigated the specificity of the Aβ_42_ protein spiked in PBS to demonstrate its feasibility for the detection of analytes in complex biofluids. As shown in Figure S5, Supporting Information, different types of biomolecules^[^ ^58^ ^]^ were added to PBS, namely immunoglobulin G (IgG), spike protein (S protein), β glucose and Na^2 +^. The concentration for all the samples tested was 1000 ng mL^−1^ and the corresponding box line plots for each sample were plotted separately. Among them, the response of the proposed TFBG biosensor to non‐specific proteins is low, proving its high specificity.

In order to further explore the effectiveness and significance of the biosensor in the biofluid where Aβ_42_ is naturally present, CSF has been chosen as a true and validated biological matrix. Therefore, we further detected Aβ_42_ in CSF of mice under the same conditions as the above experiments.

**Figure 3:**
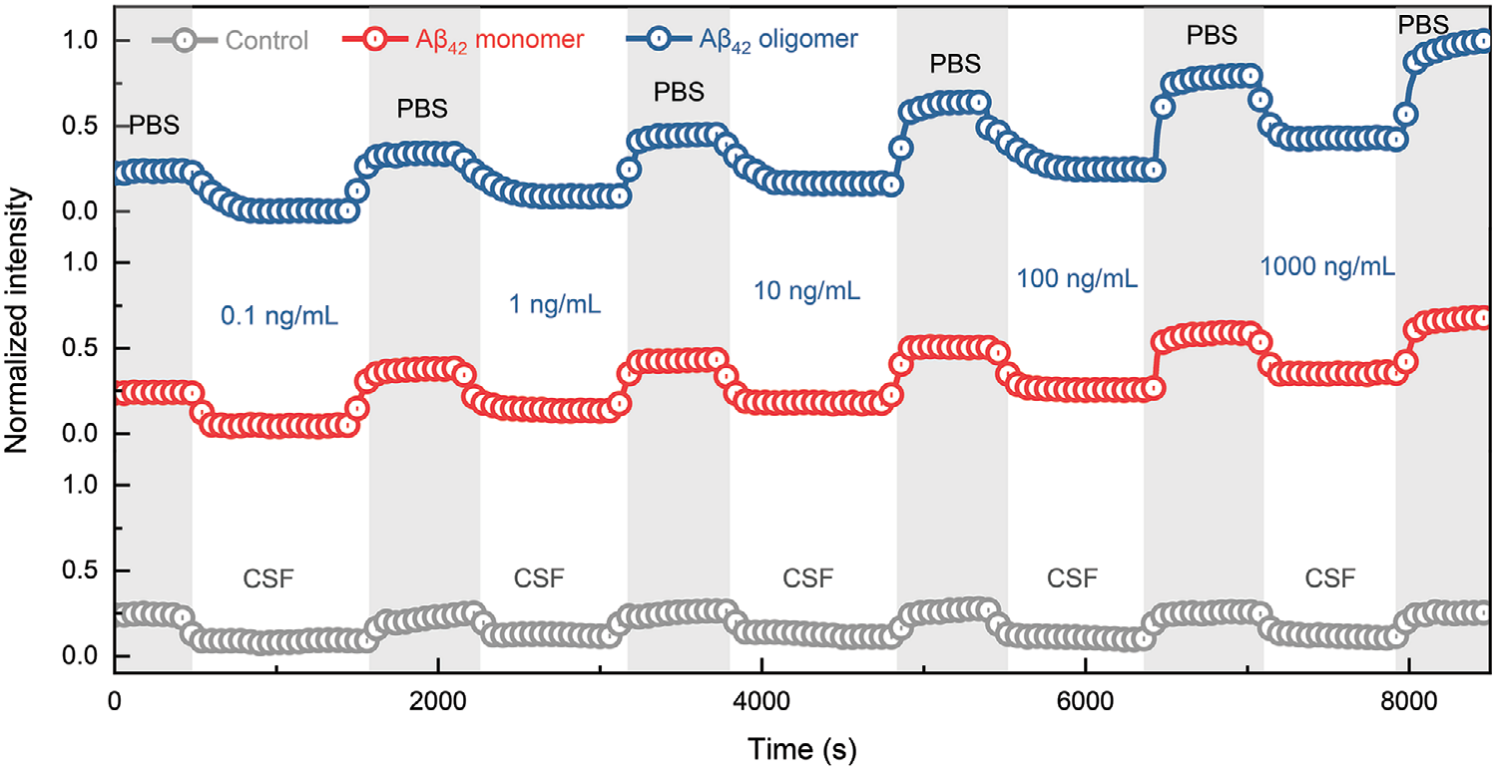
Real‐time responses of TFBG‐SPR biosensors for the detection of Aβ_42_ monomer and oligomer in CSF. Real‐time evolution of intensity curves in response to different concentrations of Aβ_42_ monomers (red dotted line), Aβ_42_ oligomers (blue dotted line), and control group with CSF without any analyte (gray dotted line).

A 1:100 dilution in PBS of CSF was chosen as the standard procedure to work with real biofluids. In fact, dilutions (1:10 or even 1:100) of complex biofluids are commonly used to evaluate biosensing platforms without having a real impact on the performances.^[^ ^59^, ^60^ ^]^ **Figure** **3** illustrates the real‐time response curve (normalized intensity vs. time) for different concentrations of Aβ_42_ monomers (red dot line) oligomers (blue dot line) respectively, spiked in 1:100 PBS‐diluted CSF, and CSF without Aβ proteins as control group (gray dot line). As it can be seen in Figure 3, the increasing trend of the light intensity of the optical signal is consistent with the results achieved when Aβ_42_ monomers and oligomers were spiked with PBS (Figure S2a, Supporting Information), which indicates the feasibility of the proposed TFBG‐SPR biosensor in a real scenario. As shown in Figure 3, compared to PBS, the light intensity gradually decreases and reaches a stable value after some minutes of the injection of the different concentrations of Aβ_42_ that guarantees a complete antibody‐antigen binding process.^[^ ^61^ ^]^ This response testifies a fast binding kinetics (the real binding interaction) without any slow binding process (like diffusion) occurred, thus underpinning the high affinity of the selected antibody‐antigen pair and the good stability of the microfluidic system as well. Conversely, the light intensity in PBS increases after the injection of Aβ_42_ concentration, but the plateau due to saturation of available binding sites is not reached given the small concentrations tested in a clinical relevant range. This can be foreseen since the greater Aβ_42_ concentration tested is 50‐fold smaller than the concentration of the anti‐Aβ_42_ antibody grafted onto the fiber surface.

It should be pointed out that that the amount of change in the optical signal (i.e., light intensity) in CSF after the injection of a specified concentration of Aβ_42_ monomers and oligomers was not always the same even if it followed an increasing trend as expected. This slight difference could stem from the morphology of Aβ_42_ oligomers that varies as they range from dimers to large multimers,^[^ ^62^ ^]^ which in turn could lead to some small dissimilarity in the molecular weight of oligomers, and hence in the induced effective RI variation. This is more evident for the lowest concentration of 0.1 ng mL^−1^ rather than the others. However, this issue would not jeopardize the performance, effectiveness and reliability of the proposed biosensing platform.

When assessing the biosensor performance, CSF was also tested for a more real and complete specificity test and as control group while detecting Aβ_42_ protein, and its signal before and after the injection exhibited a slight averaged increase in intensity in the range of 0.001 dB in replicated experiments. Although there was still very weak interaction (absorption or binding) of biomolecules with the fiber surface probably due to some protein content in CSF, the effect on the Aβ_42_ detection results is negligible since the change in the optical signal is comparable with the system noise (i.e., achieved standard deviation).

Again, as showed in Figure 3, the optical signal in PBS after the injection of a specified concentration of Aβ_42_ monomers and oligomers was not always the same and, in this case, the total change in light intensity was slightly below for Aβ_42_ monomers than that for Aβ_42_ oligomers (Figure S3, Supporting Information). Obviously, the total change in light intensity was below on average than that of showed in Figure S2a, Supporting Information when Aβ_42_ was spiked in simple PBS. The reason for this drop of the performance that happens to almost all biosensors working with real biofluids clearly stems from the increased complexity in matrix used (CSF vs. PBS) where other interfering proteins can affect the analytical capability of the biosensor.^[^ ^40^, ^63^ ^]^ However, even considering this unyielding issue, the proposed biosensing platform still enabled to detect low concentrations of Aβ_42_ in a real biological fluid from mice CSF and this feature should not be underestimated since there are very few fiber‐based biosensing platform that can currently manage this.

### 2.4 Discrimination and Reproducibility of Aβ_42_ Monomer and Oligomer

**Figure 4:**
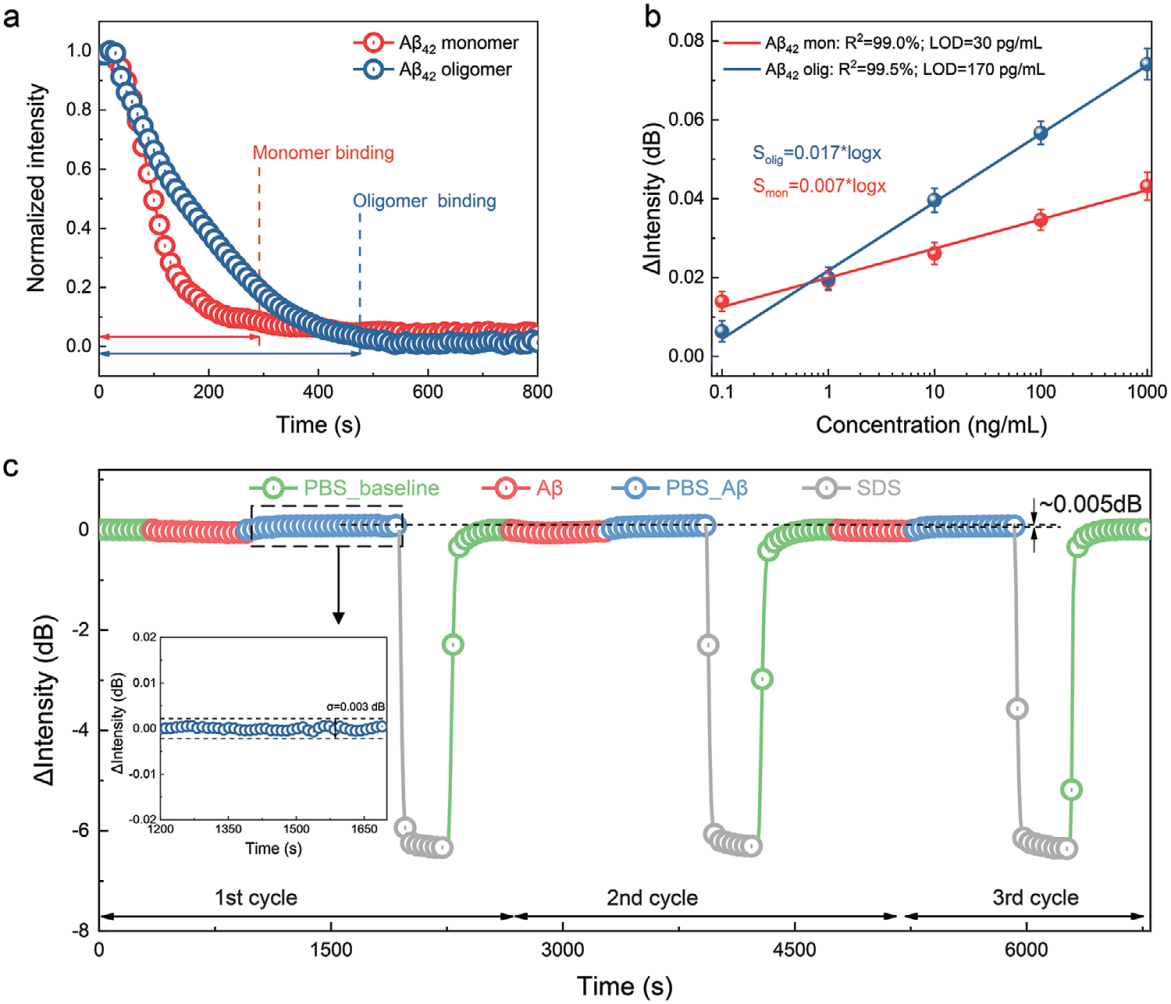
Discrimination of Aβ_42_ monomer and oligomer in CSF and the sensor's reproducibility in experiments. a) Comparative binding response in terms of relative light intensity changes when Aβ_42_ monomer and oligomer at 1 ng mL^−1^ are injected, which indicates that the relative light intensity changes of monomer and oligomer reach a stable condition, respectively; b) semi‐log dose‐response curve to detect Aβ_42_ protein for both monomers (red) and oligomers (blue); c) regeneration test using sodium dodecyl sulfate (SDS) solution on TFBG‐SPR biosensors repeated three times when a solution of 1000 ng mL^−1^ Aβ_42_ protein spiked in CSF is injected, inset: intensity fluctuation during PBS rinsing and the baseline recovering.

As a remarkable and novel breakthrough in optical biosensing, we demonstrated the possibility of discriminating between Aβ_42_ monomers and oligomers using TFBG‐SPR biosensor. In order to do this, we analyzed them from two perspectives, that is, molecular interactions and sensitivity, respectively. To make it clearer, the real‐time plots of Aβ_42_ monomers and oligomers at the concentration of 100 ng mL^−1^ were selected, as shown in **Figure** **4**a. The intensity change of Aβ_42_ monomer decreases and gradually reaches stable binding condition after red vertical line and, similarly, the intensity of Aβ_42_ oligomer more slowly reaches a stable binding condition after blue vertical line. As one might expect, the time taken for Aβ_42_ monomers to attain a stable value of the optical signal is shorter than that for oligomers. This could most likely stem from the fact that monomers have a smaller molecular weight rather than oligomers, and hence possess a less inherent hysteric hindrance with respect to oligomers. Therefore, monomer can reach the fiber surface faster and can bind to the antibody quickly. The initial binding rate (IBR) was also considered as a further analytical indicator to discriminate between monomer and oligomer. It is possible to observe from Figure S4, Supporting Information that Aβ_42_ oligomer IBR is lower than that of Aβ_42_ monomer at each concentration and the IBR value in module increases as a function of the analyte concentration.^[^ ^64^ ^]^

Figure 4b reports the dose‐response curve in semi‐log scale for both Aβ_42_ protein (red for monomers and blue for oligomers). The curve slope is slightly higher for oligomers than that for monomers given the greater molecular weight of oligomers that induces a larger variation in the effective RI measured by the optical fiber sensor. However, a high linearity is attained in both cases. By using the dose‐response curves for the biosensor used to detect Aβ_42_ monomer and oligomer in CSF (Figure S3b, Supporting Information), we further calculated the limit of detection (LOD) of 30 pg mL^−1^ for monomer and 170 pg mL^−1^ for oligomer, considering three times the standard deviation of the blank sample (0.003 dB), divided by the surface sensitivity.^[^ ^65^ ^]^ Figure S2b, Supporting Information shows the dose‐response curve for Aβ_42_ monomer and oligomer when spiked in PBS. The surface sensitivities for monomer and oligomer were 0.045 and 0.056 dB/(ng mL^−1^), respectively. Therefore, the LOD of the biosensor for the detection of Aβ_42_ monomers and oligomers in PBS is found to be 3 and 13 pg mL^−1^, respectively. As expected, given the reasons previously mentioned, the more complex the biofluid is, the worse the LOD is.

A regeneration test was also carried out to assess the reproducibility of TFBG‐SPR biosensors in view of possible reusability. As shown in Figure 4c, after the detection of Aβ_42_ from 0.1 to 1000 ng mL^−1^ with the TFBG‐SPR biosensor, a protocol involving SDS was used to regenerate the biofilm surface and then a high concentration of 1000 ng mL^−1^ of Aβ_42_ was detected again for three times. It can be seen that both the baseline after SDS washing (where the antibody and antigen have been unbound) and the baseline where the antigen binds to the antibody again are basically unchanged, which well illustrates that this TFBG‐SPR biosensor has good regeneration ability and reproducible performance. In addition, these remarkable results provide a further demonstration of the potential of SDS as regeneration solution working with antibodies.^[^ ^40^, ^66^ ^]^

### 2.5 Better Performances Comparison with Traditional Methods

**Figure 5:**
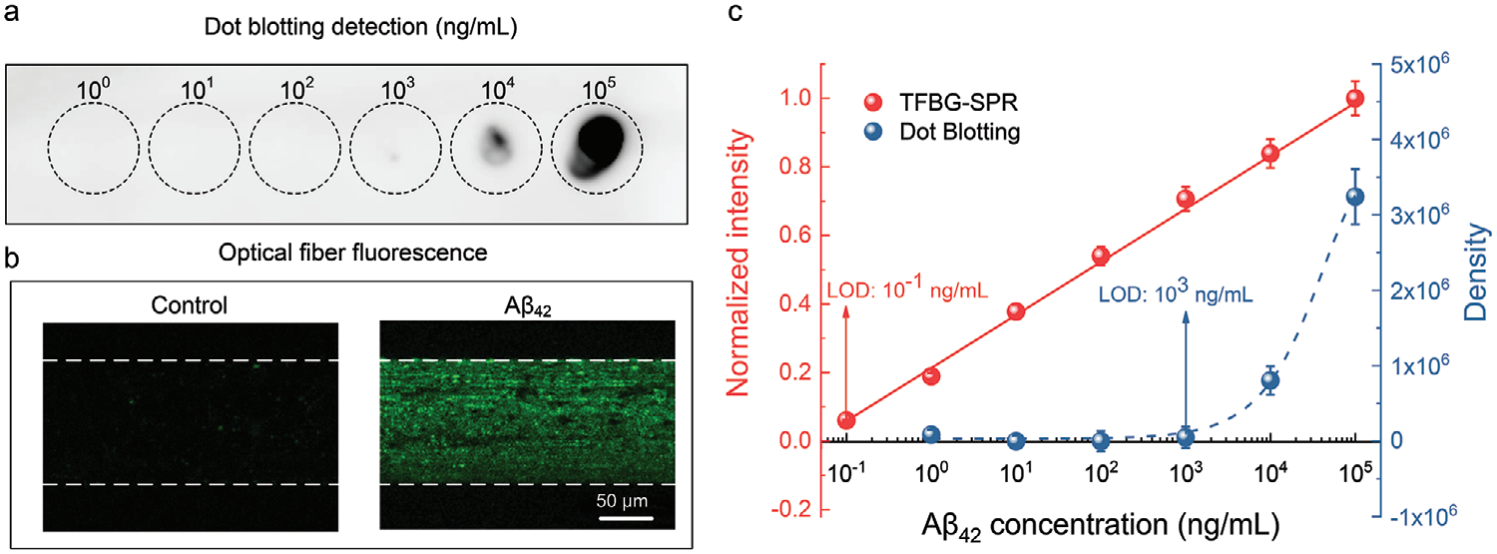
Comparison between TFBG‐SPR biosensors and traditional detection methods. a) Dot blotting of Aβ_42_; b) fluorescence detection of TFBG‐SPR biosensor in control and Aβ_42_; c) comparison of the sensitivity of TFBG‐SPR with that of Dot blotting for the detection of Aβ_42_.

To benchmark and validate our work, specificity analysis was performed in comparison with other more traditional biochemical methods, such as Dot blotting. As shown in **Figure** **5**a, from the final data obtained, the corresponding grayscale values were not observed when concentrations of Aβ_42_ was below 100 ng mL^−1^, whereas the grayscale values appeared at 1 µg mL^−1^ (evidenced in Figure 5c with a blue arrow) and gradually increased when the concentrations grew. Therefore, we can assume that the LOD of Dot blotting is around 1 µg mL^−1^. In contrast, our proposed TFBG‐SPR can detect Aβ_42_ protein at a concentration below 0.1 ng mL^−1^ (evidenced in Figure 5c with a red arrow).

To further corroborate the above experimental results and to assess the effectiveness and reliability of the antibody to specifically recognize the Aβ_42_ protein, we used immunofluorescence to detect trapped Aβ_42_ proteins on the surface of the fiber. By comparing the fluorescence response from Figure 5b where control in PBS and Aβ_42_ protein at a concentration of 10^5^ ng mL^−1^ are detailed, it can clearly be observed that there is only a background noise of fluorescence for control, while the fluorescence spreads over the functionalized surface of the fiber for Aβ_42_ protein. Therefore, it is clearly shown that the antibody can specifically recognize Aβ_42_ and, moreover, the experiment verifies the feasibility of using the TFBG‐SPR biosensor to detect the Aβ_42_ concentration. In addition, it is possible to assess that the amount of Aβ_42_ bound to the fiber surface increases with the Aβ_42_ concentration, which validates the previous experimental results.

It can be claimed that the LOD of the proposed biosensing platform consisting of a gold‐coated TFBG biosensor is 30 pg mL^−1^ for Aβ_42_ monomers or 170 pg mL^−1^ for Aβ_42_ oligomers, which is 4–5 orders of magnitude lower than the LOD achieved with conventional immunoassays‐based methods (as detailed reported in **Table** **1**). Most importantly, Dot blotting and fluorescence cannot distinguish the morphology of Aβ protein, while our proposed TFBG‐SPR biosensor can discriminate it from both sensitivity and molecular interaction, respectively, thus providing a new perspective and means to distinguish the multiple morphologies of the protein.

**Table 1:** Performance comparison of different detection methods.

Method	Sensing configuration	Target Aβ	LOD (ng/mL)	Matrix	Discrimination	Ref.
Colorimetry	Metal oxide	Monomer	16	CSF	None	^[^ ^67^ ^]^
Fluorescence	Polymer fluorescent probe	Monomer	0.77	CSF	None	^[^ ^68^ ^]^
Electrochemistry	Antibody‐aptamer electrode	Oligomer	0.4	CSF	None	^[^ ^69^ ^]^
Capillary electrophoresis	Fused‐silica capillary	Monomer	2.2	PBS	None	^[^ ^70^ ^]^
Dot blotting	Nitrocellulose sheet	Oligomer	830	PBS	None	^[^ ^71^ ^]^
Surface plasmon resonance	Optical fiber grating and microfluidic chip	Mono and oligomer	0.03∼0.17	CSF and PBS	Yes	This work

For sake of comparison with different detection methods, Table 1 gathers the LOD and distinguishing ability of different detection methods. For common detection methods, such as colorimetry,^[^ ^67^ ^]^ fluorescence,^[^ ^68^ ^]^ electrochemical,^[^ ^69^ ^]^ and capillary electrophoresis,^[^ ^70^ ^]^ optical fiber based SPR seems to have more potential for AD detection, especially for multiple markers differentiation and highly sensitive detection, which provides a novel platform for optical fiber in bioassays.

## 3 Discussion

In summary, we developed a label‐free TFBG‐SPR biosensor to achieve ultra‐sensitive detection and discrimination of the AD pathogenic factor Aβ_42_ protein. A layer of anti‐Aβ_42_ antibody is deposited onto gold‐coated TFBG by self‐assembly monolayer technique, which can bind specifically its antigen Aβ_42_. The same antibody is used to recognize the two different forms of Aβ_42_ (i.e., monomers and oligomers). Therefore, the discrimination is underpinned in terms of kinetics of interaction (response time, initial binding rate, etc.) and surface sensitivity by working in the same experimental conditions to investigate the inherent nature of both proteins. In addition, the binding kinetics of Aβ monomers to antibodies is different than that of Aβ oligomers with a faster response time and a higher IBR for monomers given their smaller hysteric hindrance and molecular weight. The surface sensitivity in response to RI changes occurring onto the functionalized surface of the fiber is slightly higher for oligomers than that for monomers and this might stem from the larger size of oligomers. All the previous parameters can remarkably be used to distinguish between different forms of Aβ proteins. The next step of this research will envisage the mixing of oligomers and monomers into a single solution and analyze the response. To this purpose, two different perspectives will be followed. The first strategy will rely on the use of two different ligands that target monomers and oligomers separately, such as antibodies sensitive to one protein type only or custom‐designed DNA aptamers, which are sensitive to monomers and oligomers separately. The second approach will be based on a pre‐screening process before the effective detection where two different types of biorecognition elements that specifically recognize monomer and oligomer are firstly designed and then linked to streptavidin. At the same time, biotin is immobilized on the functionalized surface of the fiber enabling the binding to streptavidin.^[^ ^47^ ^]^

Furthermore, the platform presented here is able to be extended to detect other causative and novel agents in Alzheimer's disease by using different engineered natural or synthetic bioreceptors, and also possesses the capability for simultaneous detection of different AD‐related biomarkers thanks to the integration with multiplexed TFBG‐ SPR biosensors. Besides, the efforts and research done in the cross‐fertilization between photonics and multidisciplinary disciplines such as biology, energy, and electrochemistry have made it possible to quantify substances for relevant applications in clinical, environmental, and industrial production, thus paving the way to translate the technology from the optical bench to the market.

True multidisciplinary efforts between photonics and biomedical groups have led to impressive clinically applications for many substances that require detection and quantification. It is these efforts that are allowing the full potential of such optical fiber sensors to be reached, and be able to exploit the extraordinary “sensitivity + specificity + reproducibility” with plasmonic fiber gratings and microfluidic chips, and the combination of other advanced sensing technologies.

## 4 Experimental Section

### Materials and Reagents

Single‐mode optical fiber with core and cladding diameters of ≈8–10 µm and 125 µm, respectively, was purchased from Corning Inc. (New York, USA). EDC, NHS, MUA, and TWEEN 20 were purchased from Sigma‐Aldrich. BSA, sodium chloride (NaCl), and β‐glucose were purchased from Shanghai Macklin Biochemical Co., Ltd., while SDS was purchased from Aladdin. The Aβ_42_ protein (beta‐amyloid (1–42) HFIP, a‐1163‐2) was purchased from rPeptide. The antibodies used for Aβ_42_ detection and dot blotting, that is, purified anti‐β‐Amyloid and 1–16 antibody, respectively, were purchased from BioLegend (803001). The secondary antibodies used for dot blotting, that is, anti‐mouse IgG and HRP‐linked antibody (1:2000) were purchased from Cell Signaling Technology (7076S). The primary antibody used for immunostaining, that is, anti‐beta amyloid 1–42 antibody (1:1000) was purchased from abcam (ab10148), while the secondary antibody used for immunostaining, that is, goat anti‐rabbit IgG (H+L) highly cross‐adsorbed antibody, Alexa Fluor 546 (1:2000) was purchased from Thermo Fisher Scientific (A‐11035). Human IgG and S protein were purchased from Beijing Bioss biotechnology Co., Ltd. CSF of mice was extracted and obtained from Laboratory of Neuroscience and Innovative Drug Research (JNU‐HKUST), Jinan University, Guangzhou. All the chemical reagents were of analytical grade. All buffers were prepared with deionized water (18.2 MΩ cm^−1^).

### Mice Cerebrospinal Fluid Collection

The 2 months old male C57BL/6J mice were purchased from Liaoning Changsheng Biotechnology co. Ltd., China. All animal experiments were carried out in accordance with the Ethics Committee on Animal Experiments at Jinan University (No: 20220812‐19). Cerebrospinal fluid was collected after modification according to this protocol.^[^ ^72^ ^]^ In brief, mice were anaesthetized intraperitoneally with 1.25% tribromoethanol and secured in a stereotaxic apparatus (RWD Life Science). Scissors were used to cut the mouse skin across the back of the neck, and then curved forceps were used to separate the musculature bluntly until exposing the cerebellar medullary cisterns without any bleeding. Using a glass capillary (sutter, #BF150‐86‐10; O.D.: 1.5 mm; I.D: 0.86 mm; length: 10 cm) to gently puncture the dura mater, leaving the capillaries in place to slowly collect cerebrospinal fluid. The cerebrospinal fluid was kept at −80 °C until use.

### Fabrication of Titled Fiber Bragg Gratings

The TFBGs were fabricated by inscribing in the core of a hydrogen‐carrying single‐mode fiber with a phase mask and a laser emitting at 193 nm (Bragg Star Industrial, Coherent, Inc.). Then an ultra‐thin gold film was deposited on the TFBGs using sputtering technology (Microtech Technology Development, China). During the sputtering process, the TFBGs needed to be rotated along its axis in order to obtain a uniform deposition of the gold film around the fiber surface. The tilt of the grating is an important parameter for selecting which group of cladding modes can be excited. Hence, the operating range of the TFBG can be adjusted by changing the tilt angle to optimize the response to certain refractive index of bio‐samples. Here, the TFBGs had a tilt of 16° and a length of 15 mm, and hence the core mode is located at ≈1590 nm and tens of cladding modes ranging from 1500 to 1580 nm are generated in the spectral comb.

### Surface Gold Coating of Optical Fiber Sensor

The gold can be successfully deposited on the TFBGs by using a standard sputtering process. In these experiments, a polaron Instruments model sputtering chamber (TRI‐S500 fiber material metal coating system) was used in the following conditions: the vacuum was obtained in the chamber starting from ambient air to a pressure of 10^−3^ Pa by pumping. The Ar gas was filled in the chamber to set the pressure as 5 Pa. Then the charged particles bombarded the Au target material into small single atom to attach to the fiber surface. A consecutive deposition process was made, with the fibers rotated alone axis by constant speed (0.5 rad s^−1^), to ensure that the whole surface was covered by Au uniformly. By controlling the deposition time, 50 nm thickness of Au was chosen to compete the coating.

### Experimental Setup

**Figure 6:**
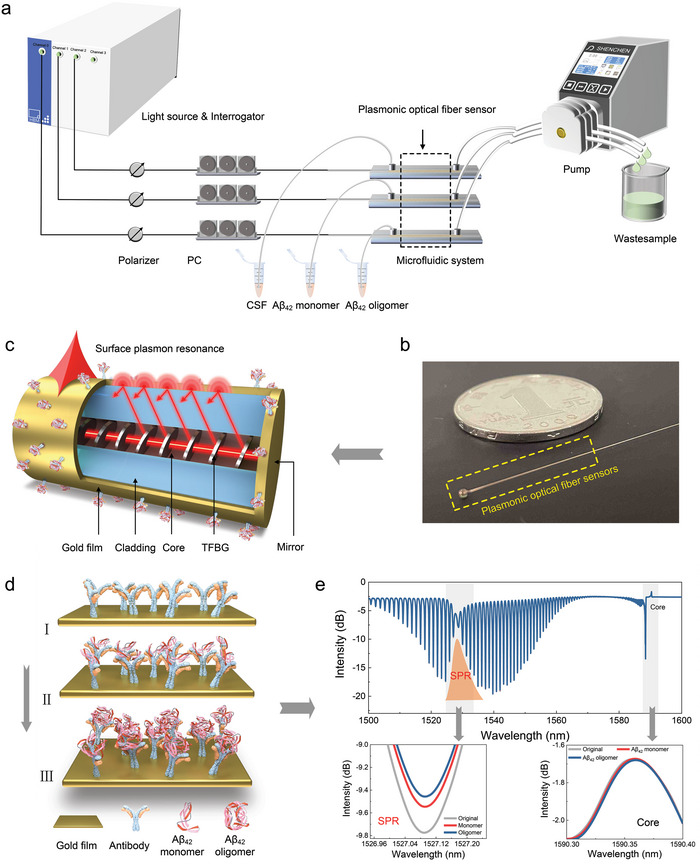
Plasmonic gold film‐coated TFBG‐based SPR biosensor for Aβ protein detection. a) Schematic diagram of the experimental setup for biosensing; b) physical map of TFBG‐SPR biosensor; c) schematic of the TFBG‐SPR biosensor sensing mechanism; d) TFBG‐SPR biosensor binds to antibodies, Aβ_42_ monomers and Aβ_42_ oligomers, respectively; e) spectral change diagram of TFBG‐SPR biosensor.

The schematic representation of the TFBG‐SPR biosensor for the detection of Aβ_42_ monomer and oligomer is shown in **Figure** **6**a, in which the photography of the sensing system is shown in Figure S6, Supporting Information. A light source (FS22 Industrial BraggMETER) with a wavelength range of 1500–1600 nm was used to launch light into TFBG to provide superfine plasmonic spectral combs. The interrogator system had a resolution of 5 pm and an adjustable sampling rate of 1 Hz–1 kHz, which made it more suitable for on‐site inspection applications. In order to adjust and orient the state of polarization of light, a linear polarizer and a polarization controller were located between the optical source and microfluidic platform. The reflection spectrum was monitored by the BraggMETER. One end of the microfluidic system was connected to a peristaltic pump to control the flow rate of the injected samples. The samples were injected into the microfluidic channels with a peristaltic pump at a speed of 4 µL min^−1^, and each concentration was detected for 15 min at room temperature. Unbound proteins were washed with PBS at a higher rate of 12 µL min^−1^. The spectra from 1522 to 1533 nm were recorded every 10 s with Labview software and detected the intensity change of one of the modes with Matlab. It should be noted that BraggMETER combines a light source and a demodulation device together, which can both transmit the light source and receive the optical signal. At the same time, it has four channels, which can realize the simultaneous detection of multiple parameters. In this experiment, channel 0 is the control group, channel 1 is used to detect Aβ_42_ monomers, and channel 2 is used to detect Aβ_42_ oligomers. It is worth mentioning that the temperature fluctuations can have an impact on the sensor response. Moreover, it can also influence in a such extent the binding interactions during a bioassay. Therefore, it turns out crucial to have an environment with a stable temperature and a system to minimize the temperature fluctuations. In this case, all the experiments were carried out at room temperature in controlled conditions (25±1 °C). However, in order to avoid any impact of temperature fluctuations on the sensor response, any change in temperature was decoupled by the core mode of the TFBG sensor by using the Bragg resonance that enabled to eliminate the temperature interference.^[^ ^47^, ^49^ ^]^

### Microfluidic System

The microfluidic system where a fiber‐optic device was embedded for sensing purposes is shown in Figure S7, Supporting Information. The microfluidic system consists of two parts with the same dimensions: 25 mm wide, 4 mm deep, and 80 mm long.^[^ ^65^ ^]^ The upper component is made up of poly (methyl methacrylate) (PMMA) and the lower component is made up of stainless steel. The fluidic channel with dimensions of 1 mm × 1 mm × 40 mm was engraved on both components in order to keep the fiber exactly in the middle of the channel, and hence where there is not any perturbation in the solution flow underpinning a laminar flow. A 0.5 mm deep V‐groove was engraved at both ends of the PMMA component to house the TFBG‐SPR biosensor. In addition, at the two ends of the PMMA channel, two stainless steel tubing were fixed and glued with an optical adhesive from the bottom to the top to be used as inlet and outlet of the sample flow. To ensure mechanical stability and to guarantee the waterproof of the device, a UV optical flexible adhesive (NOA 68, Norland Products, Inc.) was used to bond the fiber optic sensor to the edges of the V‐grooves and a Parafilm R sheet was placed between the PMMA and stainless‐steel components. It is important to note that the sensing area of the fiber device is located within the fluidic channel to allow the fiber sensor to be fully immersed by the liquid sample.

### Surface Plasmon Resonance Excitation over Optical Fiber

A photography and schematic representation of the proposed compact fiber‐optic biosensor are shown in Figures 6b and 6c, respectively. With P polarization light input, the SPR is created by the oscillation of freely moving electrons or surface plasmons on the metal surface in reaction to light absorption, and the changes in the RIs of the metal and the surrounding medium have a significant impact on the SPR signature. When the incident light polarization direction is parallel to the grating plane, and the TFBG cladding mode meets the wave vector matching with the SPs on the surface of the optical fiber gold film, the condition for the generation of SPR signature can be fulfilled. The cladding mode that meets the coupling conditions will have a high energy density on the surface of the gold film, which is exponentially attenuated in the direction perpendicular to the fiber core axis. Its transmission distance was only 250–1000 nm and it has a high interface sensitivity, so any weak change (nm scale) occurring at the gold film‐surroundings interface can be detected. Therefore, the actual sensing mechanism relies on the detection of RI changes related to any substance/molecule adsorbed or grafted on the surface of the gold film, that is to say the surface RI changes.^[^ ^42^ ^]^

### Surface Functionalization of Optical Fiber Sensor

The schematic diagram on Figure 6d depicts the immobilization of the antibody as BRE on the gold‐coated TFBG surface and the biosensing process. The SAM technology used to functionalize the fiber surface the change in the optical signal in the fiber transmission spectrum under three different conditions: antibody deposition, interaction between the antibody and the Aβ_42_ monomer and the Aβ_42_ oligomer. The sensing area of TFBG was immersed in 4 nM of MUA in absolute ethanol solution and placed in a refrigerator at 4 °C overnight, thus resulting in the spontaneous formation of an orientationally ordered, and structurally dense single‐molecule film on the gold surface, which provided gold–sulfur chemical bonds to covalently immobilize the biorecognition element. The terminal thiols group in the self‐assembled membrane on the gold surface can be covalently bound to the selected BRE. To activate carboxylic groups, the fiber sensor was immersed EDC/NHS solution (2 mM/5 mM, respectively) for 30 min. Afterward, in order to attach the BRE on the surface of the fiber, the fiber was immersed in the solution of anti‐Aβ_42_ antibody with the concentration of 50 µg mL^−1^ for 2 h to bond the anti‐Aβ_42_ to the self‐assembled monolayer of carboxyl group, and then PBS rinse for 10–15 min to remove unreacted antibodies. Finally, a solution of 1% (w/v) BSA was used for 1 h to block its non‐specific binding sites. Alongside, various concentrations of Aβ_42_ protein solutions (0.1–1000 ng mL^−1^ in steps of one order of magnitude) were prepared by spiking them in the biofluid of 1:100 PBS‐diluted CSF.

The spectral responses of proposed plasmonic TFBG to monomer and oligomer of Aβ_42_ are shown in Figure 6e. The intensity of the SPR excitation package cladding mode changes when the Aβ_42_ antibody was deposited onto the fiber surface by SAM technique and interacted with Aβ_42_ by the antigen‐antibody affinity method. Usually, the higher the number of Aβ_42_ binding, the stronger the relative intensity modulation of the narrow cladding pattern.

### Fluorescence Characterization

The fibers were divided into three groups and incubated with PBS, Aβ_42_ monomer and oligomer, respectively, for 60 min after antibody modification. Afterward, a blocking solution (4% goat serum and 1% BSA in PBS) was injected for 60 min at room temperature. Then the fibers were immunostained with a mixture of primary antibodies in a diluent (4% goat serum and 1% BSA in PBS) for 60 min at room temperature. After rinsing three times in PBS for 5 min each, fibers were incubated with fluorochrome‐conjugated secondary antibodies (lex = 561 nm, lem = 572 nm) for 120 min at room temperature in dark light condition. After rinsing three times in PBS for 5 min each in dark light condition, fibers were fixed on a microscope slide and analyzed with a laser‐scanning confocal microscope (Zeiss 700; Carl Zeiss AG, Oberkochen, Germany).

### Dot Blotting Sensing Technology

The polyvinylidene fluoride membrane (Millipore, ISEQ00010) was immersed in methanol, then dried for 20 min at 37 °C. 2 µL of Aβ samples (1–100 000 ng mL^−1^ in steps of one order of magnitude) were spotted on the membrane, then dried for 30 min at 37 °C. After washed one time with TBS‐T for 5 min, the membrane was blocked by 5% BSA in TBS‐T for 30 min at room temperature. Then washed one time with TBS‐T for 5 min, the membrane was incubated with the primary antibody for 30 min at room temperature. Then washed three times with TBS‐T for 5 min, the membrane was incubated with the secondary antibody for 20 min at room temperature. After washed three times with TBS‐T for 5 min, an enhanced chemiluminescence reagent was used to visualize immunoreactive dots. The dot intensities were quantitated by Quantity One software.

### Statistical Analysis

Data were normalized in the pre‐processing and then expressed as mean ± standard deviation after the elaboration, considering three independent repetitions (*n* = 3) of each experiment and a data set of 30 samples for each experimental point. Origin 2018 and Matlab R2018b software programs were utilized to perform the statistical analysis and create the related graphs.

## Conflict of Interest

The authors declare no conflict of interest.

## Author Contributions

L.Z., X.W., P.L., and J.X. contributed equally to this work. T.G., L.S., W.B., and F.C. conceived the key concpets, developed the theory and supervised the project. L.Z. and P.L. drafted the manuscript. X.W., W.L., and Z.L. fabricated sensors. L.Z., X.W., and J.X. performed experiments and analyzed the data. P.L. performed the animal experiments and fluorescence charaterization. X.Z., S.Z., K.L., and A.G. participated the theoretical analysis. All the authors provided critical revision of the manuscript for important intellectual content.

## Supporting information

Supporting Information

## Data Availability

The data that support the findings of this study are available from the corresponding author upon reasonable request.
