# Supplementary material for: Ultrasensitive and Multiple Biomarker Discrimination for Alzheimer's Disease via Plasmonic & Microfluidic Sensing Technologies
Source: Adv Sci (Weinh). 2024 Mar 20;11(24):2308783. doi: 10.1002/advs.202308783 (PMC11200013; doi:10.1002/advs.202308783)
Supplement: Supplementary file 1 — Supporting Information [file ADVS-11-2308783-s001.pdf]

ADVANCED SCIENCE
Open Access

Supporting Information

for *Adv. Sci.*, DOI 10.1002/advs.202308783

Ultrasensitive and Multiple Biomarker Discrimination for Alzheimer's Disease via Plasmonic & Microfluidic Sensing Technologies

Lijiao Zu, Xicheng Wang, Peng Liu, Jiwei Xie, Xuejun Zhang, Weiru Liu, Zhencheng Li, Shiqing Zhang, Kaiwei Li, Ambra Giannetti, Wei Bi, Francesco Chiavaioli*, Lei Shi* and Tuan Guo**

Supporting Information

Ultrasensitive and multiple biomarker discrimination for Alzheimer's disease via plasmonic & microfluidic sensing technologies

Lijiao Zu[1], Xicheng Wang[1], Peng Liu[2], Jiwei Xie[1], Xuejun Zhang[3], Weiru Liu[1], Zhencheng Li[1], Shiqing Zhang[2], Kaiwei Li[1], Ambra Giannetti[4], Wei Bi[5,], Francesco Chiavaioli[4,*], Lei Shi[2,*], and Tuan Guo[1,*]*

[1] *Institute of Photonics Technology, Jinan University, Guangzhou 510632, China*

[2] *JNU-HKUST Joint Laboratory for Neuroscience and Innovative Drug Research, College of Pharmacy, Jinan University, Guangzhou 510632, China*

[3] *Center for Advanced Biomedical Imaging and Photonics, Division of Gastroenterology, Department of Medicine, Beth Israel Deaconess Medical Center, Harvard University, Boston, 02215, USA*

[4] *National Research Council of Italy (CNR), Institute of Applied Physics "Nello Carrara" (IFAC), 50019 Sesto Fiorentino, Italy*

[5] *Department of Neurology, The First Affiliated Hospital of Jinan University, Guangzhou 510632, China*

[*]Correspondence and requests for materials should be addressed to Wei Bi (tbwneurodoc@jnu.edu.cn) or Francesco Chiavaioli (f.chiavaioli@ifac.cnr.it) or Lei Shi (t_shilei@jnu.edu.cn) or Tuan Guo (tuanguo@jnu.edu.cn).

S1. Real-time response for interface modifications

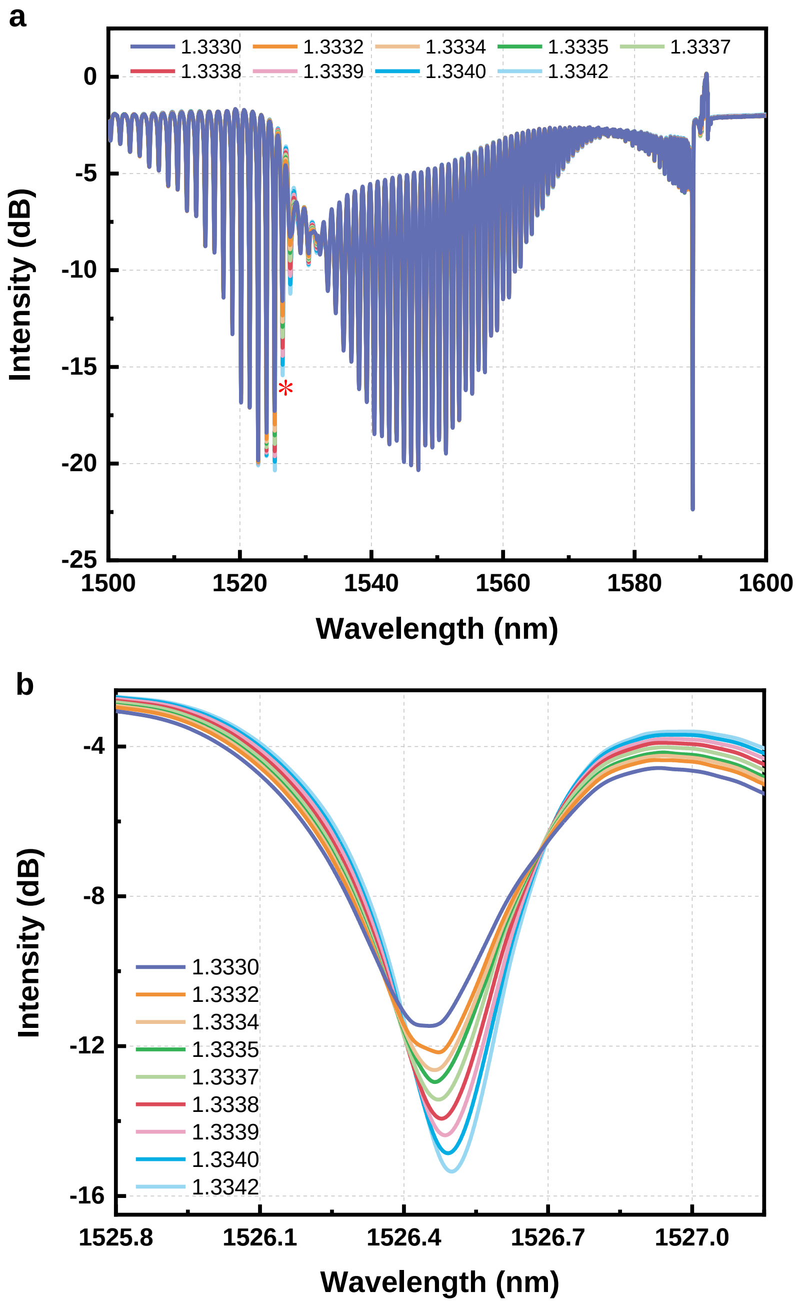

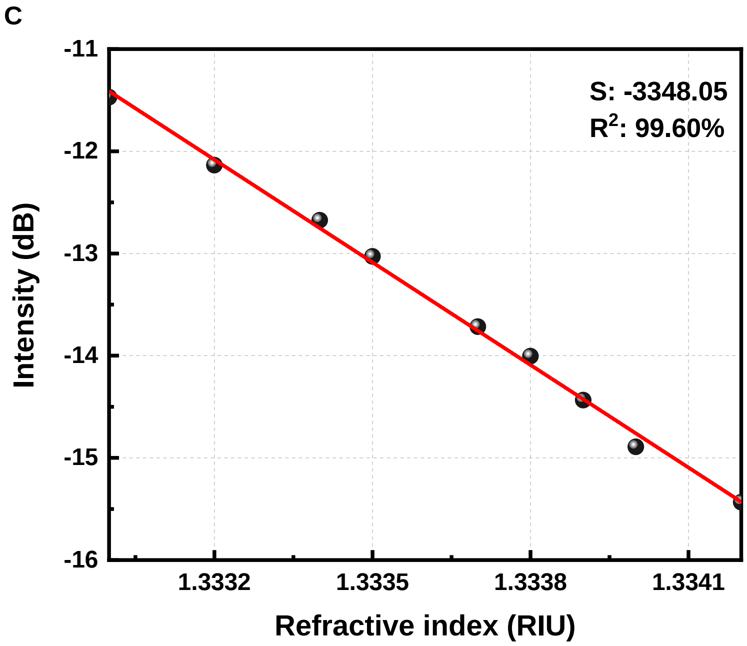

Figure S1. Optical characterization of TFBG-SPR sensor in terms of bulk sensitivity. a) Spectral response of the TFBG-SPR sensor as a function of different values of refractive index. The star symbol (*) highlights the SPR mode at the highest sensitivity; b) SPR mode amplitude changes under different refractive indices; c) response curve of the selected SPR resonance as a function of refractive index change and its linear fitting.

S2. TFBG-SPR biosensor in response to Aβ$_{42}$ monomer and oligomer in PBS

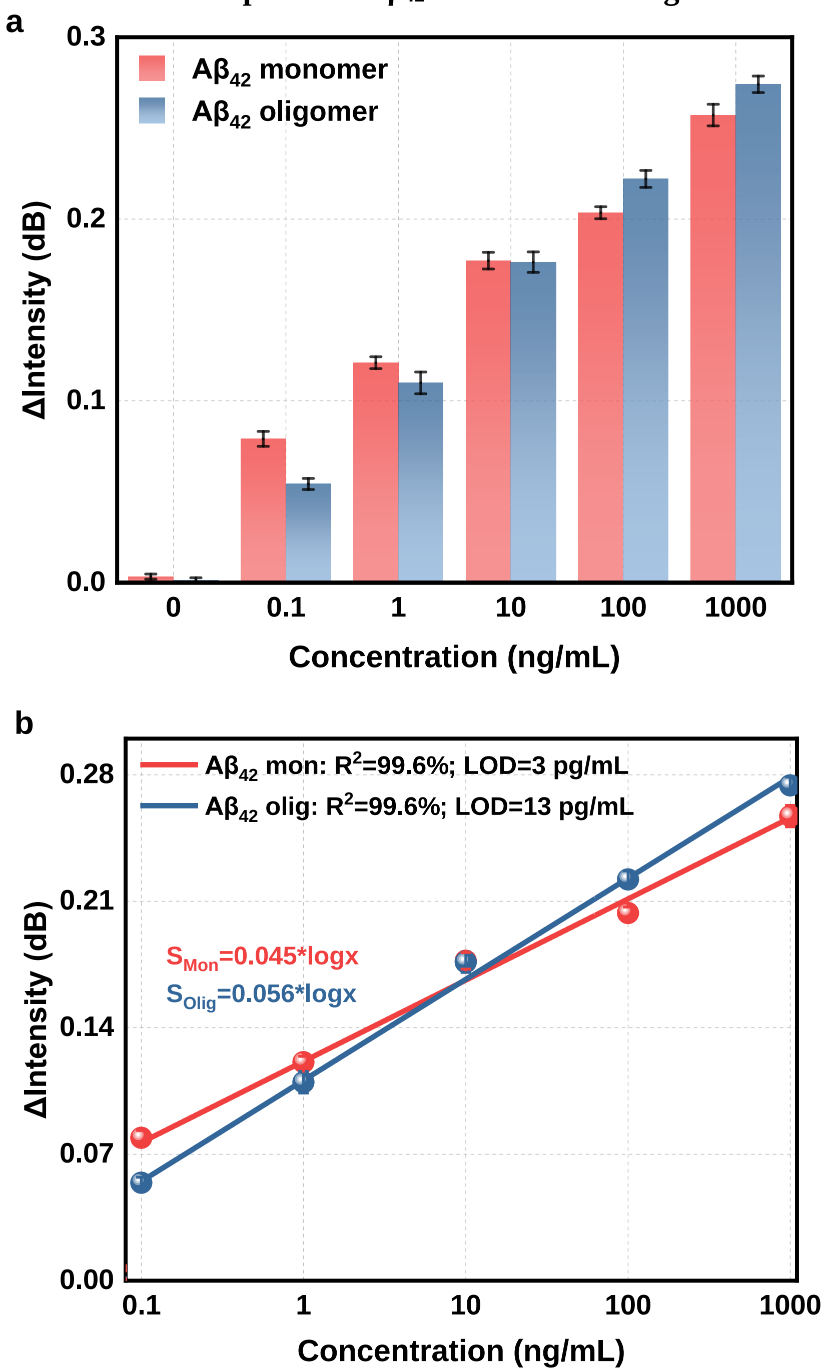

Figure S2. Dose-response curve of TFBG-SPR biosensors for the detection of Aβ$_{42}$ in PBS. a) Histogram of intensity changes of gold-coated TFBG biosensors for the detection of Aβ$_{42}$ spiked in PBS with concentrations increasing from 0 to 1000 ng mL^{-1}; b) dose-response curve of gold-coated TFBG biosensors in semi-log scale for the detection of Aβ$_{42}$ monomers (red) and oligomers (blue) in PBS and related sensitivities.

S3. TFBG-SPR biosensor in response to Aβ42 monomer and oligomer in mouse CSF

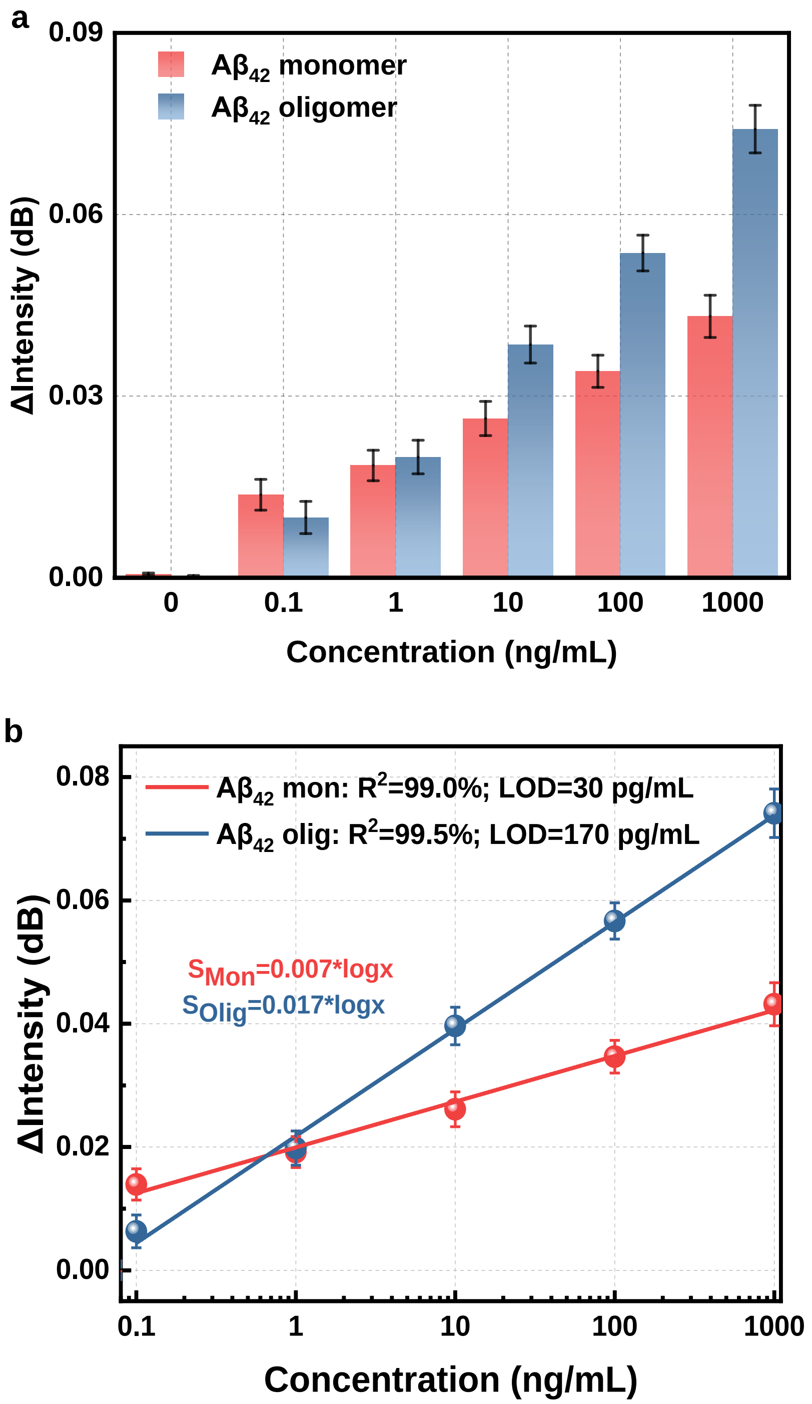

Figure S3. Dose-response curve of TFBG-SPR biosensors for the detection of Aβ42 in mouse CSF. a) Histogram of intensity changes of gold-coated TFBG biosensors for the detection of Aβ42 spiked in CSF with concentrations increasing from 0 to 1000 ng mL^{-1}; b) dose-response curve of gold-coated TFBG biosensors in semi-log scale for the detection of Aβ42 monomers (red) and oligomers (blue) in CSF and related sensitivities.

S4. Initial Binding Rate as further analytical performance indicator

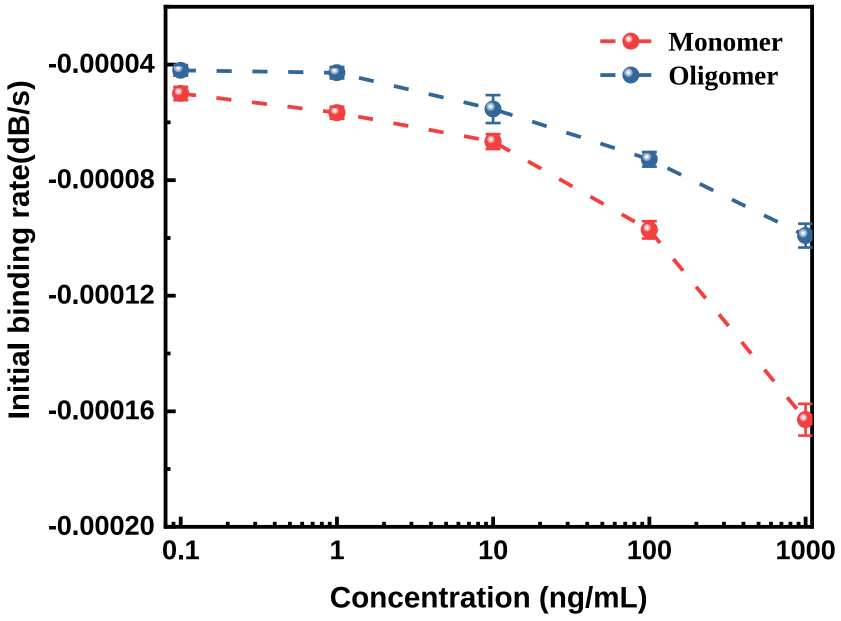

Figure S4. Dose-response curve of TFBG-SPR biosensors in semi-log scale for the detection of Aβ_{42} as a function of the initial binding rate (monomers in red and oligomers in blue). The dashed lines are the broken lines that connect the experimental points and are drawn with the purpose to provide a general view of the trends.

S5. Specificity testing with analytes spiked in PBS

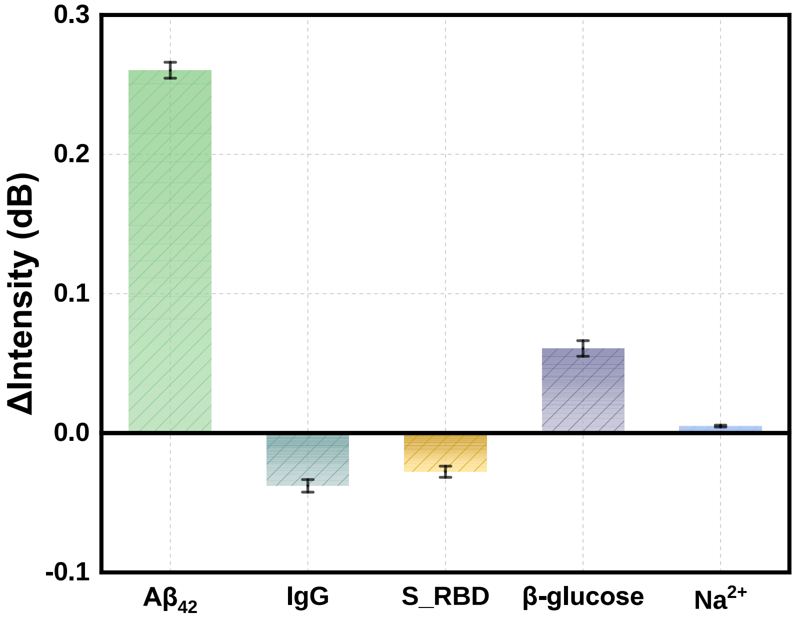

Figure S5. Specificity testing performed using different biological samples spiked in PBS with equal concentrations of Aβ_{42}, IgG, S_RBD, β-glucose and Na^{2+}.

S6. The photograph of the self-developed on bench experimental setup using TFBG-SPR biosensors

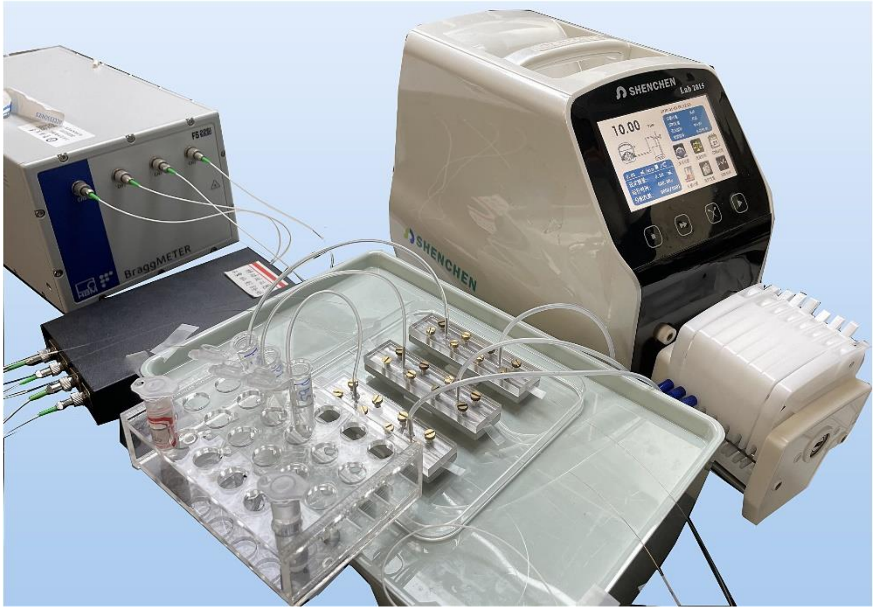

Figure S6. Photograph of the experimental platform: multichannel & microfluidic controlled TFBG-SPR biosensing system.

S7. Photograph of the single microfluidic channel

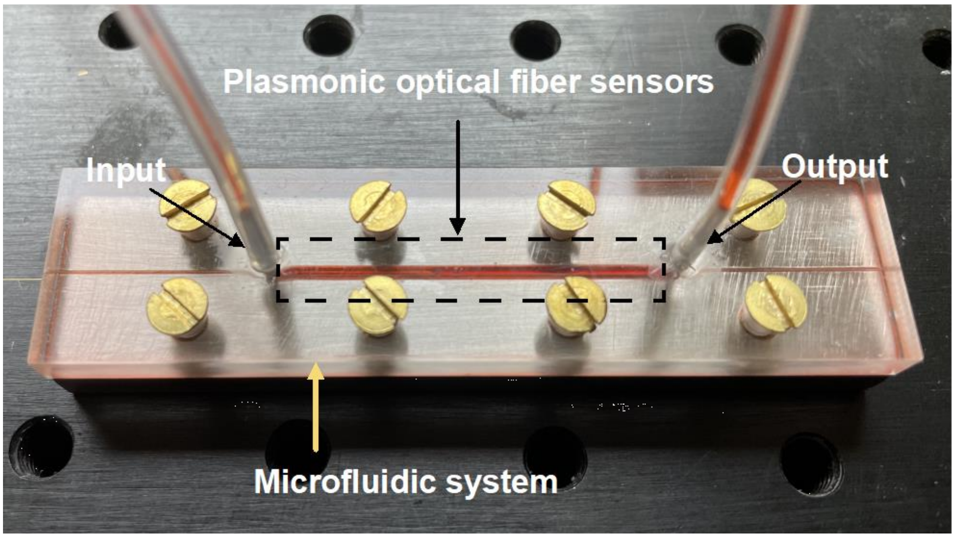

Figure S7. Photograph of the single microfluidic channel with the embedded plasmonic optical fiber sensor.
